# Association Between Vitamin D Binding Protein Gene Polymorphism (rs7041), Vitamin D Receptor, and 25-Hydroxyvitamin D Serum Levels With Prostate Cancer in Kurdish Population in West of Iran

**DOI:** 10.3389/pore.2022.1610246

**Published:** 2022-08-09

**Authors:** Mohammad Amiri, Daniel Elieh Ali Komi, Asad Vaisi-Raygani, Amir Kiani, Mahmoudreza Moradi, Mahdieh Aliyari, Zohreh Rahimi, Ehsan Mohammadi-Noori, Homayoon Bashiri

**Affiliations:** ^1^ Student Research Committee, Kermanshah University of Medical Sciences, Kermanshah, Iran; ^2^ Regenerative Medicine Research Center (RMRC), Kermanshah University of Medical Sciences, Kermanshah, Iran; ^3^ Fertility and Infertility Research Center, Kermanshah University of Medical Sciences, Kermanshah, Iran; ^4^ Medical Biology Research Center, Kermanshah University of Medical Sciences, Kermanshah, Iran; ^5^ Pharmaceutical Sciences Research Center, Health Institute, Kermanshah University of Medical Sciences, Kermanshah, Iran; ^6^ Department of Internal Medicine, Kermanshah University of Medical Sciences, Kermanshah, Iran

**Keywords:** prostate cancer, polymorphism, vitamin D receptor, vitamin D binding protein, 25(OH)-vitamin D

## Abstract

Prostate cancer (PCa) pathology has been linked to vitamin D, vitamin D receptors (VDRs), and vitamin D binding proteins (VDBPs). We sought to investigate the association between VDR rs2228570 and rs1544410 as well as VDBP rs7041 polymorphisms and serum 25-hydroxyvitamin D (25(OH)-vitamin D) levels in PCa patients. Blood samples were collected from 111 PCa patients and 150 age-matched healthy volunteers. The VDR rs2228570 T/C, rs1544410 G/A, and VDBP rs7041 T/G genotypes were determined using polymerase chain reaction-restriction fragment length polymorphism (PCR-RFLP). 25(OH)-vitamin D and PSA (Total and Free) serum levels were measured. The frequencies of VDBP genotypes T/G vs. T/T (56.5% vs. 44.5%, *p* = 0.01) according to the dominant model T/G + G/G vs. T/T (84.3% vs. 71.5%, *p* = 0.01) were significantly higher in PCa patients when compared to control group and considerably increased the risk of disease by 2.29, 1.44, and 2.13 folds respectively. Interestingly, the results demonstrated that PCa patients with the dominant model (T/G + G/G vs. T/T) of VDBP had significantly lower serum levels of vitamin D and higher serum levels of total and free PSA in comparison to the controls. Furthermore, when compared to controls, PCa patients with the dominant model T allele (T/G + G/G vs. TT) of VDBP had significantly higher vitamin D, total PSA, and free PSA concentrations. Serum levels of 25(OH)-vitamin D and rs7041 T/G polymorphism of the VDBP gene could be potential risk factors for PCa.

## Introduction

Prostate cancer (PCa) is one of the main common causes of cancer-associated deaths in men worldwide [1]. Although the cause of PCa is unknown, risk factors such as aging, race, family history, lifestyle, genetic predisposition, and environmental variables all play a role in the disease’s onset and pathogenesis [2, 3]. From a genetic standpoint, vitamin D binding protein (VDBP) is the albuminoid family’s oldest member [4] and its coding gene contains 13 exons and 12 introns and is located on the long arm of chromosome 4 (4q12-q13) [5, 6]. VDBP is a serum 2-globulin with a molecular weight of 52–59 kDa, 458 amino acids, and three unique domains [6]. The rs7041(Asp416Glu) SNP in domain III is linked to the generation of VDBP genetic isoforms [7, 8]. VDBP is also an anti-inflammatory and immunoregulatory protein that could play a role in the etiology of a variety of chronic disorders [8]. Some studies have found a direct link between VDBP levels in the blood and various malignancies, including colorectal and prostate cancers [7, 9]. VDR belongs to the nuclear receptor protein family and is found in a variety of cell types. This receptor is involved in a variety of vitamin D3 biological processes, including cell division and differentiation [10, 11]. Both tumor and normal tissues express the VDR [12]. The VDR gene has 11 exons and is located on chromosome 12 (12q13) [13]. The VDR is an important regulator of serum calcium levels. VDR appears to play a function in cell cycle control and cell apoptosis, according to evidence [14]. The SNPs rs2228570 and rs1544410 in exon 2 and intron 8 of the VDR gene, respectively, have been found to influence the risk of cancer and other disorders [15–17]. The rs2228570 polymorphism in the VDR gene is the only one that has an effect on the protein structure [18]. Vitamin D has involvement in cell proliferation, differentiation, cell cycle progression, apoptosis, and has a protective effect against cancer, diabetes, auto-immune illnesses, hypertension, and cardiovascular diseases, in addition to its well-known role in osteology [19]. Calcitriol inhibits the growth of many malignant cells by inhibiting cell-cycle progression and promoting the apoptosis [20–22]. Therefore, it may be related to lower incidence of PCa [23–25]. Calcitriol has been shown to have anti-proliferative and pro-differentiating properties on PCa cell lines [26]. Prostate cells, interestingly, can produce calcitriol from 25(OH)-vitamin D. As a result, serum 25(OH)-vitamin D levels appear to play a significant role in the onset and progression of PCa [27]. Furthermore, calcitriol and its analogs were found to have an inhibitory effect on tumor volume and metastasis [26, 28]. We performed this case-control study to investigate the relationship between 25(OH)-vitamin D serum level, VDR (rs2228570, rs1544410) and VDBP (rs7041) gene polymorphisms with the risk of PCa in a population from west of Iran.

## Materials and Methods

### Studied Population, Tests, and Obtained Data

In this case-control study, 111 PCa patients with a mean age of 67.7 ± 11.2 years old and 150 healthy controls (66.2 ± 11.3 years old) from the Kermanshah University of Medical Sciences clinic were enrolled. A full biography (medical and family) was taken from each participant and all were physically examined by a urologist and data regarding age, history of smoking, height, weight, and pretreatment testosterone levels were collected. The clinical characteristics of medical records were documented, including Gleason score, family history, and type of treatment. The levels of prostate specific antigen (PSA) in all participants were measured in order to diagnose the condition. Histopathology confirmation was used to select patients with PCa. Control subjects with PSA levels >2.5 ng/ml and the ratio of free PSA to total PSA ≤0.1 or positive digital rectal examination (DRE) were excluded. Patients having any sort of cancer or a family history of PCa were not allowed to participate in the study. The ethnic background of patients and controls was western Iranian Kurdish.

### Ethics

This research was approved by the ethical committee of Kermanshah University of Medical Sciences. We followed the guidelines approved by the Helsinki Research Ethics Committee of the Iranian Authority Ministry of Health. Written-signed consent was obtained from all included individuals.

### DNA Extraction

5 ml of peripheral blood samples were collected from all participants and genomic DNA was isolated from blood leukocytes using the phenol-chloroform method. The extracted DNA was kept at −20°C until it could be examined [29, 30].

### Detection of Genotypes

#### VDR rs2228570 T/C Polymorphism

**FIGURE 1:**
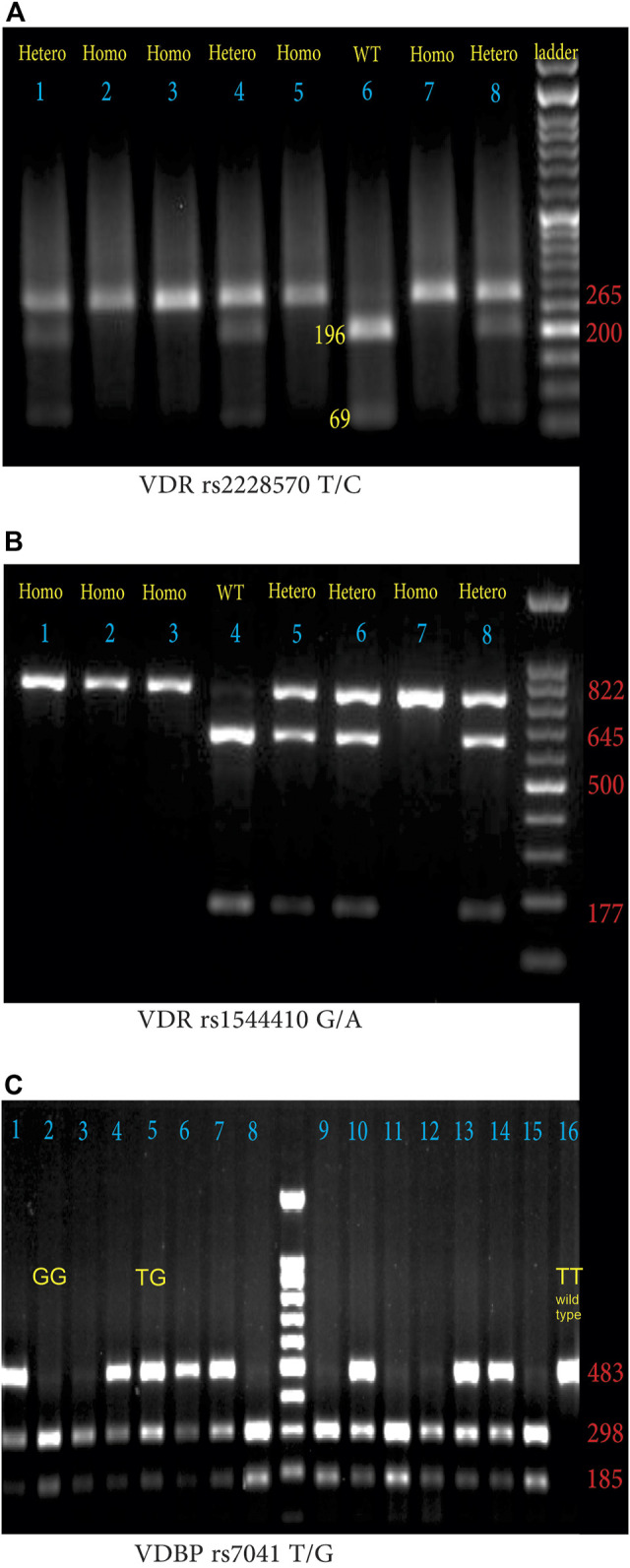
**(A)** Agarose gel electrophoresis (1.5%) pattern of VDR rs2228570 T/C digested PCR products with FOK I enzyme. From left to right lanes 2, 3, 5, and 7 indicate HOM: homozygous mutant (CC: 265 bp), lanes 1, 4, and 8 demonstrate HET; heterozygous mutant (TC: 265, 196, 69 bp); lanes 6 indicates WT; homozygous wild-type (TT: 196 bp and 69 bp). (DNA ladder = 50 bp), **(B)** Agarose gel electrophoresis (1.5%) pattern of VDR rs1544410 G/A digested PCR products with BsmI enzyme. From left to right lanes 1, 2, 3, and 4 indicate HOM, homozygous mutant (AA: 822 bp); lanes 5, 6, and 8 demonstrate HET: heterozygous mutant (AG: 822, 645, 177 bp), lanes 4 indicates WT: homozygous wild-type (GG: 645bp and 177 bp). (DNA ladder = 100 bp) **(C)** Agarose gel electrophoresis (1.5%) pattern of VDBP rs7041 T/G digested PCR products with HaeIII enzyme. From left to right lanes with two 185 and 298 bp bands indicate GG phenotype (lanes 2, 3, 8, 9, 11, 12, and 15), lanes with three bands (185, 298, and 483 bp) indicate heterozygote TG phenotype (lanes 1, 4, 5, 6, 7, 10, 13, and 14), and lane 16 with a single 483 bp band, indicates wild type TT phenotype.

The VDR genotypes were determined by PCR-RFLP with forward and reverse primers 5′- AGC​TGG​CCC​TGG​CAC​TGA​CTC​TGC​TCT -3′and 5′- ATG​GAA​ACA​CCT​TGC​TTC​TTC​TCC​CTC -3′with minor modification [31]. In brief, the PCR reaction consisted of DNA preincubation at 95°C for 4 min followed by 40 cycles at 95°C for 30 s, 68°C for 30 s, and 72°C for 1 min then cycles completed by a final extension for 5 min at 72°C. After amplification, the product (265 bp) was digested with the restriction enzyme FOK I. Wildtype TT genotype showed two fragments (196 and 69 bp) whereas, homozygous mutant CC genotype showed a 265 bp product, and heterozygous TC genotype showed three fragments (265, 196, and 69 bp) (Figure 1A).

#### VDR rs1544410 G/A Polymorphism

The forward; 5′-CAA​CCA​AGA​CTA​CAA​GTA​CCG​CGT​CAG​TGA-3′, and reverse; 5′-AAC​CAG​CGG​AAG​AGG​TCA​AGG​G-3′, primers were used to determine VDR rs1544410 G/A SNP [32]. Thermocycling condition for PCR reaction was 95°C for 5 min, followed by 30 cycles at 95°C for 1 min, 63°C for 1 min, 72°C for 1 min, with a 10 min final extension at 72°C. BsmI restriction endonuclease was used to digest the 822 bp PCR products at 37°C overnight and the digested products were separated on a 1.5% agarose gel containing ethidium bromide. The wild GG fragments were 645 bp and 177 bp, heterozygote mutant G/A fragments were 822 bp, 645 bp, and 177 bp, and homozygote AA was 822 bp as shown in Figure 1B.

#### VDBP rs7041 T/G Polymorphism

The forward 5′-AAA​TAA​TGA​GCA​AAT​GAA​AGA​AGA​C-3′ and reverse 5′-CAA​TAA​CAG​GAA​AGA​AAT​GAG​TAG​A -3′ primers were used to identify VDBP rs7041 T/G polymorphism [33]. The thermocycling condition for PCR reaction was 95°C for 5 min followed by 30 cycles at 95°C for 30 s, 55°C for 30 s, 72°C for 30 s, with a final extension at 72°C for 10 min. The amplified PCR products (483 bp) were treated with the restriction enzyme HaeIII. The lengths of digested fragments were: the TT genotype (wild type), 483 bp; the GG genotype (homozygous mutant) 298 and 185 bps; heterozygous TG genotype, 483, 298, and 185 bps (Figure 1C).

### Chemical Analyses

Total PSA, free PSA, and vitamin D concentrations in serum were evaluated using standard ELISA procedures (Monobind Ink Kit, USA), and the fPSA/tPSA ratio was calculated.

### Statistical Analysis

We calculated the allelic frequencies by the gene counting method. Pearson’s *χ* ^2^ test was used to test the difference in the distribution of the haplotypes in patients and controls. Statistical significance was assumed at *p* ≤ 0.05. The genotypes and allele frequencies of VDR rs2228570 and rs1544410 and VDBP rs7041 in PCa patients were compared to the control group using the Chi-square (*χ* ^2^) test in three different genotype models of co-dominant, the dominant/recessive, and the heterozygous. The Odds ratios (OR) and 95% confidence intervals (CI) were obtained by SPSS logistic regression (ver 16, IBM-USA). The t-test, ANOVA, and nonparametric independent sample Mann–Whitney analysis were used to compare the quantitative data.

## Results

Table 1 summarizes the demographic characteristics of the case and control groups, as well as several clinical parameters. Although there was no significant difference in age between the case and control groups, the control group’s body mass index (BMI) was considerably greater than that of PCa patients. [25.6 (14.9–43.3) vs. 24.5 (14.7–35.8) (kg/m^2^), *p* = 0.015]. In addition, the frequency of smoker PCa patients was significantly higher than smoker healthy subjects (58 (52.3%) vs. 41 (27.3%), *p* < 0.001). PCa patients have significantly lower serum levels of 25(OH)-vitamin D [16.2 (0.3–54.1) vs. 23.2 (0.3–89.8) (ng/ml), *p* < 0.001] and FPSA/TPSA ratio [0.21 (0.01–2.5) vs. 0.48 (0.01–3.5), *p* < 0.001] and higher serum levels of free PSA [14.5 (0.1–109) vs. 0.4 (0.1–1.9) (ng/L), *p* < 0.001] and total PSA [76.8 (0.4–572) vs. 0.4 (0.1–1.9) (ng/L), *p* < 0.001] compared to control group. The odd ratio (95% confidential interval) and frequencies of genotypes of VDR rs2228570 and rs1544410 and VDBP rs7041 are shown in Tables 2, 3. Statistical analysis revealed that the distribution of rs2228570 and rs1544410 genotypes had no significant differences between the case and control groups (Table 2). Table 3 shows that the frequency of T/G genotypes of the VDBP rs7041 SNP was considerably greater in PCa patients than in controls [T/G vs. T/T, (61 (56.5%) vs. 61 (44.5%), *p* = 0.015] and increased the risk of disease by 2.2 folds [2.29 (1.17–4.49, *p* = 0.015)]. The distribution of T/G + G/G genotypes according to the dominant genetic model of VDBP rs7041 SNP (T/G + G/G vs. T/T, *p* = 0.019) was significantly higher in PCa patients and increased the risk of disease by 2.13 times [2.13 (1.12–4.02, *p* = 0.019)]. The influence of the dominant model of VDBP rs7041 SNP (T/G + G/G vs. T/T) genotypes on 25(OH)-vitamin D, total PSA, free PSA, concentration, and free PSA/total PSA ratio between PCa patients and control group is presented in Table 4. Both T/T and T/G + G/G genotypes in PCa patients was strongly associated with higher concentration of total PSA [TT: 81.46 vs. 0.88 ng/L, (*p* < 0.001) and T/G + G/G: 73.58 vs. 0.89 ng/L (*p* < 0.001)] free PSA [TT: 12.69 vs.0.48 ng/L *p* < 0.001), T/G + G/G: 13.86 vs. 0.37 ng/L (*p* < 0.001)] compared to control subjects. Interestingly in presence of both T/T and T/G + G/G genotypes, the free PSA/total PSA ratio was considerably higher in the control group when compared to PCa patients [TT: 0.64 vs. 0.19 ng/L, (*p* = 0.008) and T/G + G/G: 0.43 vs. 0.22 ng/L (*p* < 0.001)]. The serum level of 25(OH)-vitamin D (ng/ml) was significantly higher in control subjects with T/G + G/G genotypes than in PCa patients [24.71 vs. 16.52, *p* < 0.001)]. The PSA serum level (Total, Free) was highly linked with the grade of disease, according to the results shown in Table 5 (*p* < 0.001). However, depending on the Gleason Score, serum levels of 25(OH)-vitamin D were similar between low and high grades of illness. We found a negative correlation between 25(OH)-vitamin D and aggressive PCa.

**TABLE 1:** The demographic characteristic and distribution of risk factors in prostatic cancer patients and control subjects in a population from the west of Iran.

Parameters	PCa (N = 111)	Control (N = 150)	*p* values
Age	67.7 ± 11.2	66.2 ± 11.3	0.613
Diabetic	No 84 (75.7%)	No 127 (84.7%)	0.068
	Yes 27 (24.3%)	Yes 23 (15.3%)	
Smoker	No 53 (47.7%)	No 109 (72.7%)	<0.001
	Yes 58 (52.3%)	Yes 41 (27.3%)	
BMI (kg/m^2^)	24.5 (14.7–35.8)	25.6 (14.9–43.3)	0.015
25(OH)-vitamin D (ng/ml)	16.2 (0.3–54.1)	23.2 (0.3–89.8)	<0.001
Total PSA (ng/L)	76.8 (0.4–572)	0.9 (0.09–3.1)	<0.001
Free PSA (ng/L)	14.5 (0.1–109)	0.4 (0.1–1.9)	<0.001
Free PSA/Total PSA	0.21 (0.01–2.5)	0.48 (0.01–3.5)	<0.001

Compared serum total PSA, free PSA, and 25(OH)-vitamin D levels and the ratio of Free PSA/Total PSA, BMI, and age between patients and controls were used two-tailed Student’s t-test.

**TABLE 2:** Odds ratio (95% confidential interval) and distribution of VDR (rs2228570) T/C and (rs1544410) G/A genotypes and alleles in patients with PCa and control subjects.

SNP	Genotype	PCa (N = 111)	Control (N = 150)
rs2228570	T/T	6 (4.1%)	6 (5.5%)
	T/C	41 (37.6%)	48 (32.7%)
		(*χ* ^2^ = 0.066, df = 1, *p* = 0.798)	
		0.85 (0.25–2.85, *p* = 0.798)	
	C/C	62 (56.9%) (*χ* ^2^ = 0.461, df = 1, *p* = 0.497)	93 (63.3%)
		0.82 (0.45–1.47, *p* = 0.497)	
		(*χ* ^2^ = 1.135, df = 2, *p* = 0.567)	
	Dominant model		
	T/T	6 (5.5%)	6 (4.1%)
	T/C + C/C	103 (94.5%)	141 (95.9%)
		(*χ* ^2^ = 0.284, df = 1, *p* = 0.596)	
		0.73 (0.23–2.33, *p* = 0.596)	
rs1544410	G/G	33 (30%)	45 (30.6%)
	G/A	61 (55.5%)	78 (53.1%)
		(*χ* ^2^ = 0.051, df = 1, *p* = 0.822)	
		1.07 (0.61–1.87, *p* = 0.822)	
	A/A	16 (14.5%)	24 (16.3%)
		(*χ* ^2^ = 0.58, df = 1, *p* = 0.81)	
		0.95 (0.64–1.4, *p* = 0.81)	
		(χ^2^ = 0.203, df = 2, *p* = 0.904)	
	Dominant model		
	G/G	33 (30%)	45 (30.6%)
	G/A + A/A	77 (70%)	102 (69.4%)
		(*χ* ^2^ = 0.011, df = 1, *p* = 0.916)	
		1.03 (0.61–1.76, *p* = 0.916)	

**TABLE 3:** Odds ratio (95% confidential interval) and distribution of VDBP (rs7041) T/G genotypes and alleles in patients with PCa and control subjects.

SNP	Genotype	PCa (N = 111)	Control (N = 150)
rs7041	T/T	17 (15.7%)	39 (28.5%)
	T/G	61 (56.5%)	61 (44.5%)
		(*χ* ^2^ = 6.016, df = 1, *p* = 0.015)	37 (27%)
		2.29 (1.17–4.49, *p* = 0.015)	
	G/G	30 (27.8%) (χ2 = 2.68, df = 1, *p* = 0.101)	
		1.36 (0.93–1.98, *p* = 0.101)	
		(*χ* ^2^ = 6.02, df = 2, *p* = 0.049)	
	Dominant model		
	T/T	17 (15.7%)	39 (28.5%)
	T/G + G/G	91 (84.3%)	98 (71.5%)
		(*χ* ^2^ = 5.54, df = 1, *p* = 0.019) 2.13 (1.12–4.02, *p* = 0.019)	
	Alleles		
	T	95 (44%)	139 (50.7%)
	G	121 (56%)	135 (49.3%)
		(*χ* ^2^ = 2.205, df = 1, *p* = 0.138)	
		1.31 (0.84–2.34, *p* = 0.138)	

**TABLE 4:** The concentration of vitamin D, total PSA, free PSA, and free PSA/total PSA with the dominant model of VDBP genotypes (T/G + G/G, T/T) comprised between PCa patients and control subjects.

Parameter	PCa (N = 111)	Control (N = 150)	*p* values
**T/T**	N = 17	N = 39	
25(OH)-vitamin D (ng/ml)	16.69	19.65	0.369
Total PSA (ng/L)	81.46	0.88	<0.001
Free PSA (ng/L)	12.69	0.48	<0.001
Free PSA/Total PSA	0.19	0.64	0.008
**T/G + G/G**	N = 91	N = 98	
25(OH)-vitamin D (ng/ml)	16.52	24.71	<0.001
Total PSA (ng/L)	73.58	0.89	<0.001
Free PSA (ng/L)	13.86	0.37	<0.001
Free PSA/Total PSA	0.22	0.43	<0.001

**TABLE 5:** Association between clinical parameters and PCa severity.

Parameter	Gleason score GS ≥ 7	Gleason score GS < 7	*p* values
25(OH)-vitamin D (ng/ml)	15.3	17.6	0.304
Total PSA (ng/L)	100.6	44.3	<0.001
Free PSA (ng/L)	20.8	6.8	<0.001
Free PSA/Total PSA	0.19	0.23	0.51

## Discussion

Although polymorphisms in the vitamin D receptor, vitamin D binding protein, and vitamin D binding protein genes have been linked to a variety of cancers, the prognostic value of VDR rs2228570 and rs1544410 and VDBP rs7041 polymorphisms in PCa is unknown. SNPs in the VDR and VDBP genes, we hypothesized, impact not only the structure and function of proteins but also vitamin D levels, potentially contributing to PCa development. The current study is the first to look at the link between smoking, VDR, and VDBP gene polymorphisms, as well as PSA and vitamin D levels in the serum, and the risk of PCa in a population from western Iran.

We demonstrated that there is a significant relationship between a low serum level of 25(OH)-vitamin D and the risk of PCa. Our findings were consistent with Ahonen et al., Atoum et al., and Deschasaux et al., who reported an inverse association between serum levels of 25(OH)-vitamin D and PCa risk among the population from Jordan, Finland, and France respectively [34–36]. Vitamin D suppresses tumor-induced angiogenesis and invasion [37]. Inconsistent with these results, Nomura et al., Ahn et al., and Travis et al. failed to indicate such an association between 25(OH)-vitamin D and PCa risk [38–40]. Their results indicated that the mean serum level of 25(OH)-vitamin D in the PCa patients (41 ng/ml, 23.6 ng/ml, and 21.4 respectively) was higher than the mean considered as deficiency (<20 ng/ml). This may be due to not excluding the individuals who took vitamin D supplements, while in our study these individuals had been excluded. As a result, inconsistencies can be caused by both environmental variables such as variation in vitamin D levels among different groups and genetic heterogenicity. We found a significantly higher frequency of TG genotype rs7041 SNP of the VDBP gene in PCa patients, which considerably increased the risk of the disease by 2.29 times. One case-control study performed by Gilbert et al. concerning associations of vitamin D pathway genes with circulating 25-hydroxyvitamin-D, 1,25-dihydroxy vitamin-D, and prostate cancer indicated that the T allele of rs7041 SNP is related to increased risk of PCa by 1.19 times among population from UK [41]. However, Maneechay et al. showed that there was a significant association between (TG/GG) genotypes of rs7041 SNP with the risk of lung cancer among the Thai population [42]. We found that PCa patients with T/G + G/G genotypes of rs7041 SNP had decreased serum levels of 25(OH)-vitamin D compared to control subjects. Approximately 90% of 25(OH)-vitamin D is bound to VDBP in blood circulation, so the serum concentrations of 25(OH)-vitamin D are affected by this protein [43]. Some studies showed a direct association between VDBP polymorphisms and 25(OH)-vitamin D serum concentration [43, 44]. In addition, the functional mutation in the VDBP gene may affect the anti-inflammatory and immunoregulatory properties of protein and increased cancer susceptibility. Our research revealed that neither rs2228570 nor rs1544410 SNPs of the VDR gene were relevant to PCa risk among the population from the west of Iran. Our results are consistent with some previous studies [35, 45]. Moreover, we found that the distribution of different genetic models of rs2228570 and rs1544410 SNPs (dominant, co-dominant, and recessive) were similar to both PCa and control groups. Although some studies reported a relationship between rs2228570 and rs1544410 polymorphisms with risk of PCa [46, 47]. As well, Ahn et al. found that the presence of polymorphism in or near the 3′ untranslated region of VDR along with decreased levels of vitamin D may be associated with the incidence of PCa among American men [48]. These contradictory results may be due to other factors that affect PCa incidence and development such as race, genetic heterogenicity, environmental factors, and sample size. In summary, our results showed that both smoking and VDBP T/G genotypes of rs7041 SNP significantly increase the risk of PCa. According to the dominant model T/G + G/G vs. TT genotypes of the VDBP gene, rs7041 SNP increases the risk of prostate cancer among the population from the west of Iran. We found that serum concentration of 25(OH)-vitamin D, total PSA, and free PSA in PCa patients carrying T/G + G/G vs. TT genotypes of VDBP rs7041 was significantly higher than the healthy subjects. Although, neither VDR rs2228570 nor rs1544410 was related to PCa. These findings should be explored in large prospective studies.

In conclusion, the frequencies of T/G genotypes of VDBP rs7041 SNP were significantly higher in PCa patients compared to control subjects [T/G vs. T/T, (61 (56.5%) vs. 61 (44.5%), *p* = 0.015] which was associated to increase the risk of disease by 2.2 folds [2.29 (1.17–4.49, *p* = 0.015)]. Moreover, we report an association between T/T and T/G + G/G genotypes in patients and the concentration of total PSA when compared to control subjects [TT: 81.46 vs. 0.88 ng/L, (*p* < 0.001) and T/G + G/G: 73.58 vs. 0.89 ng/L (*p* < 0.001)] free PSA [TT: 12.69 vs.0.48 ng/L *p* < 0.001), T/G + G/G: 13.86 vs. 0.37 ng/L (*p* < 0.001)]. Finally, we reported that in presence of both T/T and T/G + G/G genotypes, the free PSA/total PSA ratio was considerably higher in the control group when compared to PCa patients [TT: 0.64 vs. 0.19 ng/L, (*p* = 0.008) and T/G + G/G: 0.43 vs. 0.22 ng/L (*p* < 0.001)].

Finally, from a clinical point of view, the presence of mutations in the VDBP gene in PCa patients, and decrease in the serum level of vitamin D as well as the increase in tPSA and fPSA levels, may increase the risk of PCa onset, therefore, determining the genotypes of these genes in different populations and linking them with the onset of PCa parallel to measuring serum PSA and vitamin D levels. Enriching the diet with vitamin D supplements may reduce the risk of PCa in susceptible individuals.

## Data Availability

The raw data supporting the conclusions of this article will be made available by the authors, without undue reservation.

## Abbreviations

PCa, prostate cancer; PSA, prostate specific antigen; RFLP, restriction fragment length Polymorphism; VDBP, vitamin D binding protein; VDR, vitamin D receptor; WT, Wild type.
